# Headache in juvenile myoclonic epilepsy

**DOI:** 10.1007/s10194-011-0332-6

**Published:** 2011-03-25

**Authors:** Christoph J. Schankin, Jan Rémi, Ira Klaus, Petra Sostak, Veronika M. Reinisch, Soheyl Noachtar, Andreas Straube

**Affiliations:** Department of Neurology, University of Munich Hospital, Großhadern, Marchioninistr. 15, 81377 Munich, Germany

**Keywords:** Juvenile myoclonic epilepsy, Headache, Migraine, Prevalence

## Abstract

The objective of this study was to assess the prevalence of and risk factors for primary headaches in juvenile myoclonic epilepsy (JME). Headache was classified in 75 patients with JME using a questionnaire, and its prevalence was correlated with the literature on the general population and clinical data. Headache was present in 47 patients. Thirty-one had migraine [20 migraine without aura (MO), 11 migraine with aura (MA)]. Fourteen patients with migraine had tension-type headache (TTH) in addition. Sixteen had only TTH. Comparison with the general population revealed a significantly higher prevalence of migraine (RR 4.4), MO (3.6), MA (7.3) and TTH (3.4) in JME. Risk factors for migraine and MO were female gender and for MA family history of migraine in first-degree relatives. Migraine and MA were associated with fairly controlled generalized tonic clonic seizures, MO with absences. Together with its strong genetic background, JME appears to be an attractive homogenous subtype of epilepsy for genetic research on migraine.

## Introduction

Both primary headache disorders and epilepsies are chronic neurological pathologies with episodic manifestations [1]. Beyond their high prevalence in the general population, Ottman and Lipton [2] have demonstrated that migraine and epilepsy are often comorbid. Risk factors (positive family history, comorbid depression) or triggers (alcohol, menses, and irregularity of sleep) as well as prophylactic drugs (valproate, topiramate) are shared by both. This suggests that migraine and epilepsy may have some common pathophysiological mechanisms. Proposed mechanisms include an increased excitability of the cortex accounting for the increased risk of migraine and epilepsy [3] or seizures being triggered by migraine attacks, as the term migralepsy of the International Classification of Headache Disorders (ICHD-II) [4] suggests. It is well known that seizures can trigger secondary headache attacks as well (postictal headache, ICHD-II 7.6.2) [4, 5].

Juvenile myoclonic epilepsy (JME) is a generalized epilepsy syndrome [6] with a prevalence of 4–10% of all patients with epilepsy [7]. It manifests typically in the second decade [8]. The main seizure types are myoclonic jerks, generalized tonic-clonic seizures and, less frequently, absences [9]. The syndrome has a strong genetic background with at least 40% of patients presenting a positive family history [10]. Intensive genetic studies could identify genes associated with JME coding for various ion channels, but also acetylcholine receptors, lysosomal membranes, and for the regulation of apoptosis (review in [11]). Typical interictal EEG-findings are generalized polyspikes and generalized spike and wave discharges [12]. Despite being generalized, these EEG findings may have a lateralized maximum in about one-third of patients [13], and photosensitivity can be recorded in 30–90% of patients [14].

In this work, we analysed the correlation between JME and headache. Using a retrospective approach, we studied the prevalence of interictal headaches in a group of patients with JME who had presented to our outpatient epilepsy clinic. Headaches were characterized according to the ICHD-II [4] and correlations were sought between the characteristics of the headache and the clinical manifestations of JME.

## Patients and methods

### Study population

Patients with JME were retrospectively identified by searching our electronic medical records from 1999 to 2008 for the keywords juvenile myoclonic epilepsy, JME and orthographically related terms. All patients who had been given the diagnosis of JME according to the criteria of the International League Against Epilepsy (ILAE) were included [15]. A standardized letter was sent to these patients, which obtained written informed consent and included instructions stating that the study was interested in comorbidities of epilepsy. The patients were asked to give information on personal details (age, gender, family history of migraine, migraine in first-degree relatives, family history of JME, and JME in first-degree relatives) and medication to be chosen from the following list: anti-epileptic drugs, anti-pain medication (non-opioid pain medication and triptans), anti-depressants, and anti-hypertensive drugs (beta blockers, ACE inhibitors, AT1 antagonists). They were asked to report a temporal relation between their headache attacks, if present, and their seizures: no relation, headache before, during or after the seizure. The self-administered German language headache questionnaire should only be completed for the headaches unrelated to seizures to avoid a confusion of the primary headaches with the secondary headache postictal headache. For this, headache starting within 3 h after the seizure was assessed to be postictal headache [4]. This questionnaire had been validated previously according to the ICHD-II [4] for migraine, including migraine without and with aura (MO and MA), chronic migraine, episodic and chronic tension-type headache (TTH), medication overuse headache and trigeminal autonomic cephalgias [16, 17]. According to the ICHD-II each distinct type of headache that a patient had was separately diagnosed and coded resulting probably in more than one diagnosis per patient [4]. Seizure activity was taken from the patient’s personal charts and was based on the frequency of the preceding 6 months. We categorized seizure frequency as seizure-free, as rare (less than 1 per month), or as frequent (more than 1 per month).

The frequency of migraine, MO and MA in an age- and gender-matched control group was estimated using the data published by Lampl et al*.* [18]: in a first step, the number *n* of patients of each sex and age group (16–29, 30–49 and older than 50) was identified in our patients. Second, the prevalence *P* of each sex and age group (16–29, 30–49 and older than 50) was obtained from the literature [18]. Finally, the expected number of patients with migraine, MO and MA in the virtual control group was calculated by multiplying *n* by *P.* Similarly, the expected frequency of subjects with TTH in Munich (a large city in southern Germany) was calculated according to the recent prevalence data of the DMKG headache study [19] using the mean prevalence of TTH in Dortmund (a large city in western Germany) and Augsburg (a small city in southern Germany). This mean prevalence was chosen for Munich to normalize for differences according to location (southern vs. western Germany) and city size (large vs. small). When calculating the expected frequency of headache for the same number of virtual members of the general population, the absolute number of MA was too low for statistical analysis (rounded 1, data not shown). In order to achieve more realistic numbers for MA, we thus decided to double the size of the virtual normal population.

### Evaluation of EEG

All original EEG data were retrieved from our database and re-evaluated by an experienced electroencephalographer (J.R.) after blinding for seizure semiology and headache history. Interictal and ictal epileptic discharges were identified and classified as generalized sharp wave complexes, focal spikes, generalized polyspikes and photoparoxysmal reactions.

### Statistical analysis

Statistical analysis of clinical data was performed using SPSS 16.0 for Windows (SPSS Inc., Chicago, IL, USA). Categorical and continuous variables were analysed using Chi-square or Fisher’s exact test (in case of expected values <5) and two-sample *t* test to identify significant correlations. If appropriate, data are presented as mean ± standard deviation (SD) or as relative risk (RR). The level of significance was set at 0.05 in all cases.

## Results

### Study population and frequency of headache

122 patients with JME were identified from our database and were sent the questionnaire. Of these, 75 returned the completed questionnaire, resulting in a return rate of 61%. The mean age was 33.4 ± 12.4 years. 57% of the patients were women. The median age of seizure onset was 15 years. The seizure semiology was as follows: myoclonic jerks were present in all patients, generalized tonic-clonic seizures in 66 (88%), and absences in 29 (39%). EEG was done in 48 patients. Although all patients were receiving anti-epileptic treatment, only 25 (52% of 48) had no epileptiform patterns. The remaining had generalized sharp-wave complexes, focal spikes, generalized polyspikes, photoparoxymal reactions, or a combination. To address the possibility of a selection bias due to the return rate of 61%, respondents and non-respondents (*n* = 47) were compared for epilepsy characteristics and parameters known to be associated with headache occurrence. No significant difference was found between both groups for gender (females: 43 of the respondents vs. 23 of the non-respondents, *p* = 0.43), age (mean 36 ± 12 vs. 33 ±12, *p* = 0.24), anti-epileptic medication, and seizure type or frequency (data not shown).

In our group of patients with JME 47 (63%) indicated a recurrent interictal headache. The self-administered headache questionnaire identified 31 (41%) patients as having migraine. Of these, the questionnaire identified one patient with an additional trigeminoautonomic cephalgia (cluster headache) and 14 (19%) who also had headache of tension type (TTH). One of the latter also had medication overuse. 16 (21%) patients had only TTH, resulting in a total of 30 (40%) patients with TTH. 29 (39%) had episodic TTH and one (1%) chronic TTH. The migraine could be specified as MO in 20 patients and MA in 11. In respect of migraine frequency, five migraine patients had chronic migraine (one indistinguishable from medication overuse headache) (Table 1). The remaining 28 patients (37%) had no headache.

**Table 1:** Prevalence of headaches in JME

	JME	General population	*p*	RR
	No. (%)	No. (%)		
Total number	75	150		
Gender (female = 1)	43 (57%)	86 (57%)	1	
Age (mean ± SD)	33.4 ± 12.4 (n.a.)	32.6 ± 12.5 (n.a.)	0.66	
Migraine	31 (41%)	14 (9%)	<0.001*	4.4
Migraine without aura	20 (27%)	11 (7%)	<0.001*	3.6
Migraine with aura	11 (15%)	3 (2%)	<0.001*	7.3
TTH	30 (40%)	29 (19%)	0.001*	3.4
Both, migraine and TTH	14 (19%)	n.a.		
Episodic TTH	29 (39%)	n.a.		
Chronic TTH	1 (1%)	n.a.		
Chronic migraine	5 (7%)	n.a.		
MOH	2 (3%)	n.a.		
TAC	1 (1%)	n.a.		

The prevalence of headache in an age- and gender-matched general population was estimated from the literature [17, 18]. Statistics: Chi-square, Fisher’s exact test (in case of expected values < 5), * p < 0.05

*RR* relative risk

Of the 47 patients with headache, 33 were able to state which began first, headache or JME: headache came first in 10 patients (30% of 33), JME came first in 13 patients (~40%) and both started at the same age in the remaining 10 (30%). Of the 11 patients with MA, 6 remembered the beginning of their disorders: JME came first in 3 and both appeared at the same age in the other 3.

In addition, 18 (24%) patients also experienced headache in close temporal relation to a seizure. Of these, one reported headache prior to a seizure and 13 after a seizure. The remaining 4 had either headache prior and after (in 2) or during and after (in 2) a seizure.

Table 1 further shows the expected frequencies of headache in the virtual control population estimated from the data published by Lampl et al*.* [18] for migraine and by Pfaffenrath et al*.* [19] for TTH. Both migraine (*p* < 0.001) and TTH (*p* = 0.001) were significantly more frequent in the patients with JME with a relative risk (RR) for migraine of 4.4, for MO of 3.6, for MA of 7.3, and for TTH of 3.4.

### Intrapersonal and seizure-associated risk factors for headache

Statistical analysis of intrapersonal parameters identified female gender as a risk factor for migraine (*p* = 0.01, RR 1.6) and MO (*p* = 0.03, RR 1.7), and a family history of migraine in first-degree relatives as a risk factor for MA (*p* = 0.02, RR 5.3), as shown in Table 2 (only relevant data are shown for the purpose of clarity).

**Table 2:** Intrapersonal, medication- and seizure-associated risk factors for migraine, MO, MA and TTH

	Migraine versus no migraine				Tension-type headache versus no tension-type headache				Migraine without aura versus no migraine				Migraine with aura versus no migraine			
	Yes (*n* = 31)	No (*n* = 44)	*p*	RR	Yes (*n* = 30)	No (*n* = 45)	*p*	RR	Yes (*n* = 20)	No (*n* = 44)	*p*	RR	Yes (*n* = 11)	No (*n* = 44)	*p*	RR
Female gender	23	20	0.01*	1.6	20	23	0.18		15	20	0.03*	1.7	8	20	0.18	
Family history of migraine	10	8	0.16		8	10	0.66		6	8	0.29		4	8	0.23	
First degree relative	7	3	0.08		6	4	0.19		3	3	0.37		4	3	0.02*	5.3
Medication																
Non-opioid pain medication	19	14	0.01*	1.9	18	15	0.02*	1.8	13	14	0.01*	2.0	6	14	0.16	
Triptans	2	0	0.17		1	1	1.00		0	0	n.a.		2	0	0.04*	n.a
Seizures																
Absence <1 month	0	1	1.00		0	1	1.00		0	1	1.00		0	1	1.00	
Absence >1 per month	6	2	0.06		6	2	0.05		5	2	0.03*	5.5	1	2	0.50	
GTC <1 month	3	6	0.73		4	5	1.00		2	6	1.00		1	6	1.00	
GTC >1 per month	6	1	0.02*	8.5	3	4	1		3	1	0.09		3	1	0.02*	12.0

Only relevant data are shown (no correlation was found for age, family history of JME, the intake of migraine prophylactic anti-epileptic drugs, valproic acid, topiramate, beta blockers, or antidepressants, and the absence of seizures, in general, or the presence of myoclonic jerks). Statistics: Chi-square, Fisher’s exact test (in case of expected values < 5), two-sample *t* test, * p < 0.05

*GTC* generalized tonic-clonic seizure, *RR* relative risk

Patients with migraine, MO and TTH significantly more often reported taking non-opioid pain medication and patients with MA more often used triptans.

Patients with good seizure control (i.e. seizure-free or <1 seizure/month) did not show a correlation with the occurrence of any kind of headache. Migraine and MA were present significantly more often in patients reporting >1 generalized tonic-clonic seizure/month (*p* = 0.02, RR 8.5 and *p* = 0.02, RR 12.0) and MO in patients with >1 absence/month (*p* = 0.03, RR 5.5). TTH was more frequently found in patients with >1 absence/month (*p* = 0.05).

The EEG findings generalized sharp wave complexes, focal spikes and generalized polyspikes were equally distributed in all headache groups when compared to patients with JME but no headache (data not shown for the purpose of clarity). Photoparoxysmal reactions did not prevail in patients with migraine (1 patient with photoparoxysmal reactions out of 21 patients with migraine who had EEG), MO (1 of 17 with MO), MA (0 of 4 with MA) and TTH (1 of 20 with TTH) when compared with the respective non-headache group: photoparoxysmal reactions were present in 3 of 27 patients without migraine who had EEG (*p* = 0.62 for migraine, *p* = 1.00 for MO and MA) and in 3 of 28 patients without TTH (*p* = 0.63).

## Discussion

In our study, the prevalence of migraine (41%, RR 4.4) and especially MA (15%, RR 7.3) is higher in patients with the homogenous epilepsy subgroup of JME than expected from studies on the general population [18, 19]. For TTH, the prevalence of 40% (RR 3.4) in JME is also significantly higher than expected from our control population [19], but it is similar to the numbers reported elsewhere (34% in [20], 38% in [21]). The data in the literature on headache in epilepsy are in line with our findings. However, the prevalence of migraine and especially MA was even higher in our patients with JME. In respect of unselected epilepsy, a prevalence of migraine between 8 and 24% [22–25] was found. Syvertsen et al*.* [26] reported headache in 52% of patients with unselected epilepsy. This prevalence was similar to our study. Migraine, however, was less frequent (only 20%). Clarke et al. [27] found a migraine prevalence of 15% in children with the homogenous epilepsy subtype rolandic epilepsy in contrast to 7% in non-epilepsy probands. Since JME has a strong genetic background (positive family history of seizures in at least 40% [10]), headache and especially MA in JME, but maybe not in the general population, might also have such a strong genetic background. We suggest that our findings support using the relationship of JME and MA to further study the genetics of both disease entities. Although there are a lot more people with MA than JME, we believe that the co-occurrence of epilepsy and migraine in patients with JME might be based on shared pathophysiological mechanisms. Cortical spreading depression (CSD) is thought to be a key mechanism in the pathophysiology of both MA (e.g. [28]) and MO [29]. Therefore, the high prevalence of MA in JME might be related to CSD starting more often in patients with JME than without JME. This is consistent with the hypothesis that epileptic foci and CSD might facilitate each other [30]. It is thus possible that epileptic discharges with or without additional cortical epileptic signs or symptoms might stimulate the onset of CSD, resulting in an activation of the trigeminovascular system and, finally, in headache [31]. Parisi et al. [32] demonstrated the relevance of such a mechanism in a case report on a patient with photosensitive occipital lobe epilepsy and migraine attacks triggered by intermittent photic stimulation. A headache attack with migraine phenotype in patients with epilepsy thus might be associated with otherwise clinically silent seizures, calling into question the rigid differentiation between interictal and ictal headache. In contrast, photosensitivity was not associated with the occurrence of headache in our patients with JME.

The association of headache with otherwise clinically silent seizures might further be an explanation for our finding that migraine, in general, and MA, in particular, were more often present in patients with more than one seizure/month. Special caution is required in the interpretation of this data due to the low number of patients in the individual groups. However, a population-based study with 1,656 participants demonstrated similar results with 5/11 (45%) subjects with active epilepsy and only 4/28 (14%) with epilepsy in remission having migraine [33]. Since we did not perform EEG during headache in our patients, this possible association of headache attacks with seizures was not tested, but might be interesting for future studies. One consequence would be that an EEG during headache should be aimed for, especially in patients with epilepsy, but also in migraine patients without known epilepsy. A complementary explanation for the correlation of migraine with seizure frequency would be a different genetic background in patients with migraine and JME than in patients with JME alone, meaning that it is more difficult to treat epilepsy in patients with additional migraine.

Our finding that female gender and positive family history of migraine are risk factors for migraine, MO and MA were expected from migraine epidemiology. The higher intake of non-opioid pain medication in the migraine and MO groups and of triptans in patients with MA can be interpreted as the self-treatment of the headache attack. The alternative interpretation that headache is produced by the pain medication is unlikely, as only one patient was diagnosed with medication-overuse headache.

Since our cross-sectional study demonstrates only correlations, it does not allow a conclusion on the causative relationship between headache and JME to be drawn. In other words, it neither argues for headache being caused by JME nor for JME being caused by headache. Interestingly, a recent case–control study from Iceland addressed the question of a chronological interrelation of migraine and epilepsy and was able to identify a 8.1-fold increased risk for patients with MA of developing epilepsy later in life [34]. In our study, the sequence headache–epilepsy could only be established in 6 of 11 patients with MA and the interpretation is problematic due to the retrospective study design. None of these 6 patients had MA prior to the occurrence of epileptic seizures, i.e. the sequence headache–epilepsy was different from that expected according to the data from Ludvigsson et al. [34]. Although the number of patients was very low, this might point to a unique characteristic of MA in JME in contrast to unselected epilepsy.

### Limitations

One limitation of our study is that we compared our patients to a virtual population of limited sample size instead of a study-specific control group. However, we believe that our approach was accurate since (a) our findings are supported by the data in the literature as discussed above, (b) both reference studies were from an ethnic population (Austrian and Southern Germany) that was almost the same as ours and (c) the study by Lampl et al*.* [18] provided prevalence data split for age and gender allowing the estimation of an age- and gender-matched control group. Another limitation of the data is the return rate of 61% for the questionnaire. If patients with JME and headache returned their questionnaire more frequently than patients with JME without headache, a false high prevalence of headache would result. This scenario, however, is unlikely since non-respondents differed neither in demographic nor in clinical epilepsy characteristics from respondents. Furthermore, even if all patients who failed to respond were headache-free, the prevalences would be: 25% (31/122) for migraine, 16% (20/122) for MO, 9% (11/122) for MA, and 25% (30/122) for TTH. Although this could not be tested for significance, these frequencies are still higher than in our control population, but are similar to the prevalence in patients with unselected epilepsy [24]. Nonetheless, a false high prevalence cannot definitely be excluded, necessitating a prospective study using face-to-face interviews for the characterization of the headache disorder to confirm our data.

In summary, we have demonstrated a higher prevalence of MA (and to a lesser degree also of migraine, in general, and TTH) in patients with JME than in the general population and in patients with unselected epilepsy. This suggests a common, so far unknown, pathophysiological mechanism of JME and migraine. JME is a homogeneous epilepsy syndrome and seems to be a good candidate for headache research in epilepsy.
